# *Salvia miltiorrhiza* Bunge Ameliorates Benign Prostatic Hyperplasia through Regulation of Oxidative Stress via Nrf-2/HO-1 Activation

**DOI:** 10.4014/jmb.2308.08053

**Published:** 2023-10-31

**Authors:** Young-Jin Choi, Nishala Erandi Wedamulla, Seok-Hee Kim, Mirae Oh, Kang Sik Seo, Jeong Su Han, Eun Joo Lee, Young Ho Park, Young Jin Park, Eun-Kyung Kim

**Affiliations:** 1 Department of Food Science and Nutrition, Dong-A University, Busan 49315, Republic of Korea; 2 Department of Health Sciences, the Graduate School of Dong-A University, Busan 49315, Republic of Korea; 3 Department of Food Science and Technology, Faculty of Animal Science and Export Agriculture, Uva Wellassa University, Badulla 90000, Sri Lanka; 4 Grassland and Forages Division, National Institute of Animal Science, Rural Development Administration, Cheonan 31000, Republic of Korea; 5 Curome Bioscience Co., Ltd., Suwon 16506, Republic of Korea; 6 Healthism Corporation, Cheongju 28160, Republic of Korea; 7 Department of Family Medicine, Dong-A University College of Medicine, Busan 49315, Republic of Korea; 8 Educational Major, Graduate School of Education, Dong-A University, Busan 49315, Republic of Korea; 9 Nutrinomics Lab. Co., Ltd., Busan 49315, Republic of Korea

**Keywords:** Benign prostatic hyperplasia, oxidative stress, *Salvia miltiorrhiza* Bunge, androgen receptor signaling, Nrf-2/HO-1 signaling

## Abstract

Oxidative stress is a key factor in the pathogenesis of benign prostatic hyperplasia (BPH) that leads to inflammation. This study aimed to evaluate the ameliorative effects of *Salvia miltiorrhiza* Bunge extract (HLT-101) on BPH through the regulation of oxidative stress and inflammation. A testosterone propionate (TP)-induced BPH rat model was orally administered HLT-101 (20, 40, or 80 mg/kg), and its effects on oxidative stress- and inflammation-related gene expression were examined. Further, HLT-101 was assessed for its effect on reactive oxygen species (ROS) levels and Nrf-2/HO-1 signaling pathways in BPH-1 cells. HLT-101 decreased testosterone-induced excessive free radical production and inflammatory factor activation. Moreover, HLT-101 treatment significantly decreased the intracellular ROS level in the TNF-α and IFN-γ treated BPH-1 cells through the activation of Nrf-2. In addition, HLT-101 treatment inhibited the NF-κB pathway and androgen receptor (AR) signaling, which is highly linked to the pathogenesis of BPH. Therefore, HLT-101 has the potential to be an effective treatment reagent for BPH because of its ability to reduce inflammation and oxidative stress via Nrf-2/HO-1 signaling.

## Introduction

Benign prostatic hyperplasia (BPH) is a common prostate disorder affecting middle-aged and older people [1]. It is characterized by enlarged prostate glands that can cause various symptoms in the lower urinary tract [2]. These symptoms can range from mild to severe, the most common being an increased frequency of urination, a weak urine stream, and difficulty in starting or stopping urine flow. Although the exact causes of BPH are still not fully understood, oxidative stress has been identified as a contributing factor, and increased levels of oxidative stress have been reported in patients with BPH. Studies have also reported that the severity and presence of BPH correlate with oxidative DNA damage in the prostate transition zone [3]. BPH has a significant impact on the quality of life of individuals, as it can lead to restricted mobility and disability [4]. However, BPH is treatable. Treatment options range from lifestyle modifications to medications and/or surgery [5]. It is commonly prescribed to treat BPH with alpha-blockers and 5-alpha-reductase inhibitors (5ARIs) [6]. Alpha-blockers inhibit the α1-adrenergic receptors and can ease the lower urinary tract symptoms (LUTS) through the relaxation of bladder muscles. However, they do not shrink the prostate, which is an essential problem [7]. In prostate tissue, 5ARI is used to prevent testosterone from being converted to DHT [8]. This can reduce the prostate size by preventing DHT from binding to the androgen receptors and stimulating prostate growth. Although 5ARIs are effective, they require long-term use and are associated with side effects including problems, appetite loss, and impotence.

The etiology of an enlarged prostate remains unclear. It is known that the periurethral zone is affected by BPH due to proliferation of prostatic stroma and epithelial cells [11]. One of the factors contributing to this condition is the increased proliferation of stromal and epithelial cells in the prostate as well as a decrease in apoptosis [12]. Studies have revealed that oxidative stress-associated prostate tissue damage may cause compensatory cellular proliferation, leading to hyperplastic growth. Oxidative stress increases under inflammatory conditions owing to the increased production of reactive oxygen species (ROS) [13]. Similarly, prostatic inflammation leads to the generation of several free radicals, including inducible nitric oxide (iNOS), reactive nitric species, and reactive oxygen species (ROS). iNOS activates reactive nitrogen species, leading to cellular damage. Moreover, oxidative stress modulates the imbalance between cell proliferation and death by altering the cellular DNA. This induces programmed cell death, leading to hyperplastic transformation. Additionally, oxidative stress activates the transcription factor NF-κB [14]. A major role in the development of the prostate is played by dihydrotestosterone (DHT), which is produced by the prostate's 5 alpha-reductase enzyme from testosterone [15, 16]. After DHT has been synthesized into the prostate, it binds to the androgen receptor (AR) and forms a complex with it [17], which is known as DHT-AR complex [17]. DHT-AR complex translocate to the nucleus, where it interacts with androgen-responsive elements (AREs) [18]. AREs function as promoters or enhancers of transcription of AR target genes [19]. As part of this process, DHT stimulates the production of growth factors and inflammatory mediators, such as iNOS and Cox-2 [20].

The NF-κB signaling pathway is involved not only in cell inflammation but also in cell proliferation and growth. NF-kB and androgen signaling both regulate the expression of genes that are important for the development of BPH [21, 22]. Both molecules regulate the transcription of genes involved in cellular proliferation and inflammation. By regulating these pathways, NF-kB and androgen signaling can affect BPH progression. Nuclear factor erythroid 2-related factor 2 (Nrf-2) regulates inflammation in the body [23, 24]. It activates the body's antioxidant and detoxification systems, which help reduce inflammation [25]. Nrf-2 is a transcription factor activated by androgens and plays an important role in regulating cell proliferation and apoptosis in BPH [21, 26, 27]. Additionally, androgen signaling is known to be involved in the development of BPH; therefore, understanding how it interacts with Nrf-2 could help in the development of BPH treatments.

*Salvia miltiorrhiza* Bunge is native to China and has been used in traditional Chinese medicine for centuries [28]. The main components of *S. miltiorrhiza* Bunge are fat-soluble diterpene compounds including cryptotanshinone (CT), tanshinone I, IIA and IIB, dihydrotanshinone, isotanshinone I and II, isocryptotanshinone, tanshinol, rosmarinic acid, lithospermic acid, and salvianolic acid A and B [29-32]. It has anti-inflammatory [33], antioxidant [34], and anti-cancer [33-35] effects. Thus, it is currently being studied for its potential therapeutic applications. However, there is no clear evidence of the effectiveness of *S. miltiorrhiza* Bunge in BPH through the regulation of oxidative stress via Nrf-2 and AR signaling. Therefore, this study aimed to investigate whether *S. miltiorrhiza* Bunge could improve BPH symptoms by regulating oxidative stress.

## Methods

### Preparation of *S. miltiorrhiza* Bunge Extract (HLT-101)

HLT-101 was obtained from Healthism Corporation (Republic of Korea). The root of *S. miltiorrhiza* Bunge was extracted with ethanol. The extracted solution was concentrated, and dried for preservation at room temperature.

### High-Performance Liquid Chromatography (HPLC) Assay

The concentration of cryptotanshinone in HLT-101 was determined by HPLC using an Agilent 1260 series system (Agilent Technologies, Germany) with a C18 column [Phenomenex Synergi 4 μ Hydro-RP 80A (4.6 mm × 250 mm I.D.), Phenomenex, Inc. USA]. The mobile phase was a mixture of methanol and water (80:20, v/v), with isocratic elution at a flow rate of 0.8 ml/min. Column effluent was monitored at 270 nm. The injection volume was 20 μl.

### Materials

Welgene (Republic of Korea) provided the RPMI 1640 medium and fetal bovine serum (FBS). Penicillin/streptomycin (P/S) was obtained from Gibco (USA). Testosterone propionate was provided by the Tokyo Chemical Industry Co. (Japan). DHT and finasteride (Fi) were purchased from Sigma-Aldrich (USA). Human TNF-α and IFN-γ were purchased from R&D Systems (USA). Antibodies against β-actin, steroid receptor coactivator-1 (SRC-1), prostate-specific antigen (PSA), AR, proliferating cell nuclear antigen (PCNA), HO-1, iNOS, Cox-2, Bax, Bcl-2, VEGF, EGF, b-FGF, and glyceraldehyde-3-phosphate dehydrogenase (GAPDH) were purchased from Santa Cruz Biotechnology (USA). Antibodies against Nrf-2, NF-κB(p65), p21, cyclin D1, cyclin D2, and cyclin-dependent kinase (CDK)4 were purchased from Cell Signaling Technology (USA). Antibodies against PSA, 5AR2, and IGF-1 were purchased from Abcam (UK). Horseradish peroxidase (HRP)-conjugated anti-rabbit IgG, HRP-conjugated anti-mouse IgG, FSD 594-conjugated anti-rabbit IgG, and FSD 488-conjugated anti-mouse IgG were purchased from BioActs (Republic of Korea).

### DPPH Radical Scavenging Activity

Following dispensing HLT-101 (100 μl) at various concentrations into 96-well plates, 1,1-Diphenyl-2-picryl-hydrazyl (DPPH) solution (0.15 mM, 100 μl) was dispensed and incubated at room temperature for 30 min. After standing for 30 min, absorbance was recorded at 540 nm using a microplate reader. Extinction activity was calculated as a percentage using the following equation:

DPPH radical scavenging activity = [A _control_- A _sample_]/A _control_ × 100

### Metal Chelating Scavenging Activity

Metal chelating activity of HLT-101 was determined by mixing various concentrations of HLT-101 (50 μl) with 5μl of 2 mM FeCl_3_·6H_2_O. After incubation, 5 mM ferrozine (15 μl) was added and incubated for another 10 min. Following the incubation absorbance was measured at 562 nm. Metal chelating activity was determined by the following formula:

Metal chelating scavenging activity = [A _control_- A _sample_]/A _control_ × 100

### Animal Experiments

Male Sprague-Dawley rats (*n* = 56, 10-weeks-old) were obtained from Nara Biotech Co., Ltd. (Republic of Korea). A rat cage was carefully set up under the following conditions: 23–25°C, 40–60% relative humidity, and a daylight–dark cycle of 12 h with a 40–60% cycle alternating day and night every 2 h. There was an ad libitum supply of water and food. Animal care and experimental procedures were approved by the Dong-A University Animal Care and Use Committee (DIACUC-21-06). A one-week adaptation period was followed by anesthetization with phenobarbital (50 mg/kg), scrotal skin incision, suturing of the vas deferens, and removal of the testes and epididymis in order to eliminate endogenous testosterone secretion. The control (Con) group underwent incision and suturing of the scrotal skin. After 3 days, subcutaneous injections of testosterone propionate (TP) (3 mg/kg/day), dissolved in corn oil, were given for a period of four weeks to induce BPH. When TP was administered, HLT-101 (20, 40, or 80 mg/kg) was orally administered daily for 4 weeks. In the Fi group, Fi was administered orally for four weeks at a dose of 1 mg/kg/day. After the final injection, the rats were anesthetized with pentobarbital (50 mg/kg, intraperitoneal injection), their prostate was harvested, and the prostate weights (g) were measured. A portion of the ventral prostate lobe was excised, fixed in 10% formaldehyde, and subjected to histological analysis. The remaining ventral prostate lobes were placed in liquid nitrogen, stored at -80°C, and used for Western blot experiments.

### Serum Concentrations of DHT

A DHT ELISA kit (SunLong Biotech Co., China) was used to determine the concentration of DHT in the serum. Absorbance was measured at 450 nm using a spectrophotometer.

### Hematoxylin and Eosin Staining

The ventral prostate lobes fixed with 10% formaldehyde were embedded in paraffin. A Leica microtome (RM 2135; Wetzlar, Germany) was used to cut the sections to 4 mm thickness. Prior to staining with hematoxylin for 5 min and washing with water for 5 min, the sections were deparaffinized and hydrated. After staining with eosin for 30 s, the de-hydrated sections were cleared with xylene and mounted. A Leica DMi8 Research Inverted Phase microscope was used for histological examination. The epithelial thickness of the prostate and proportion of the lumen area were measured using ImageJ 1.47v software (National Institutes of Health, USA).

### Masson’s Trichrome Staining

Prostate tissue sections (4 μm) were deparaffinized and hydrated using a common xylene alcohol series. Nuclei were counterstained with Weigert’s hematoxylin solution for 5 min. After thorough washing with distilled water, the cells were stained for 10 min with Biebrich scarlet-acid fuchsin solution (Sigma-Aldrich). After washing with distilled water, the sections were treated with 1% phosphomolybdic acid solution (Sigma-Aldrich) solution for 10 min. The samples were rinsed with water for 1 min and stained with an aniline blue solution (Sigma-Aldrich) for 5 min. For observation, the sections were cemented using mounting medium, followed by rapid dehydration with 95% alcohol and absolute alcohol. We examined three areas of each sample at a magnification of 50 ×.

### Immunohistochemistry

A xylene-alcohol series was used to deparaffinize and hydrate sections (4 μm). For antigen retrieval, 0.01 M citrate buffer (pH 6.0) was used; sections were microwaved for 10 min, incubated at room temperature for 10 min, and rinsed in distilled water for 10 min. Treatment with 3% H_2_O_2_ quenched the endogenous peroxidase activity. After treating the cells with goat serum, the sections were incubated overnight at 4°C with anti-iNOS, anti-Cox-2, or anti-Nrf-2 antibodies. Mayer's hematoxylin was used to stain and count all sections after treatment with the secondary antibody for 1 h.

### Western blotting

To lyse cells and extract proteins, LNCaP and BPH-1 cells were lysed from prostate tissue with radioimmuno-precipitation assay (RIPA) lysis buffer. Prostate tissue was homogenized using a bead grinder after adding RIPA lysis buffer. Afterwards, the supernatant was obtained by sufficiently homogenizing it with a vortex and then centrifuging it at 4°C and 13,000 rpm (15,928 g) for 20 min. To estimate the protein concentrations, a bicinchoninic acid assay was conducted. A sodium dodecyl sulfate-polyacrylamide gel electrophoresis (SDS-PAGE) was used for protein separation. A sodium dodecyl sulfate-polyacrylamide gel electrophoresis (SDS-PAGE) was used for protein separation. Proteins were blocked for 1 h at room temperature in phosphate buffered saline with 0.05%TWEEN 20 (PBST) supplemented with 5% skim milk. At 4°C, the membrane was incubated with the primary antibody overnight. AffiniPure IgG bound to HRP was added to the membrane and incubated for 1 h, followed by three washes with TBST. A HRP substrate (Advansta Inc., USA) was used to image the chemiluminescent membrane using the ChemiDoc imaging system (Azure c300, Azure Biosystems, USA). ImageJ 1.47v software was used to quantify the chemiluminescence intensity of the protein signal.

### Cell Culture

In this study, LNCaP cells were provided by Korean Cell Line Bank (Republic of Korea) (KCLB number: 21741). BPH-1 cells (SCC256) were purchased from Merck (USA). In a CO_2_ incubator at 37°C, the cells were cultured in RPMI 1640 supplemented with 10% FBS and 100 mg/ml P/S. LNCaP cells were seeded into 6-well plates (approximately 1 × 10^6^ cells per well) in RPMI 1640 supplemented with 10% FBS and 100 mg/ml P/S. After 24 h, the LNCaP cells were co-treated with TP (100 nM) and HLT-101 (5, 10, or 20 μg/ml) for 24 h. As a positive control, cells treated with Fi (1 g/ml) were used. The cells were then collected for western blot analysis. In addition, no effect on LNCaP cell viability was observed with HLT-101 at any concentrations (data not shown).

BPH-1 cells were seeded in 6-well plates (approximately 2 × 10^6^ cells/well) in 2 ml of RPMI 1640 supplemented with 10% FBS and 100 mg/ml P/S. BPH-1 cells were incubated for 24 h in RPMI 1640 medium containing TNF-α (10 ng/ml), IFN-γ (10 ng/ml), and HLT-101 (5, 10, or 20 μg/ml). The cells were collected for western blotting and immuno-fluorescence analyses. Meanwhile, HLT-101 treatment resulted in a significant and dose-dependent decrease in BPH-1 cell viability (data not shown).

### Immunofluorescence

BPH-1 cells were incubated for 24 h in FBS-free RPMI 1640 medium containing TNF-α (10 ng/ml), IFN-γ (10 ng/ml), and HLT-101 (5, 10, or 20 μg/ml). The expression of Cox-2, iNOS, and NF-κB was visualized using immunofluorescence. Briefly, BPH-1 cells (approximately 5 × 10^3^ cells/well) were seeded in an 8-well slide chamber (SPL Life Science Co., Republic of Korea). Cells were fixed in ice-cold methanol and washed three times with PBS. This was followed by permeabilization with 0.1% Triton X-100 for 15 min. After blocking with 5%normal goat serum for 1 h, slides were incubated with Cox-2, iNOS, and NF-κB antibodies (1:300 dilution) overnight at 4°C. Following three PBST washes, the slides were incubated for one hour with goat anti-rabbit IgG, FSDTM 594 (1:1,000 dilution), and FSDTM 488-conjugated goat anti-mouse IgG. DAPI (four',6-diamidino-2-phenylindole) mounting medium was added to the slides and coverslips were glued on. A Zeiss 700 confocal microscope (Oberkochen, Germany) was used to capture the images.

### Statistical Analysis

Data are shown as mean ± standard error of the mean. The data were analyzed with the GraphPad Prism 9 software (ver. 9.4.1 USA). Multiple group evaluations of the means were performed using one-way analysis of variance (ANOVA) and Scheffe's multiple comparison post hoc analysis. *p* < 0.05 was considered to be statistically significant.

## Results

### Active Compounds in HLT-101

**Fig. 1:**
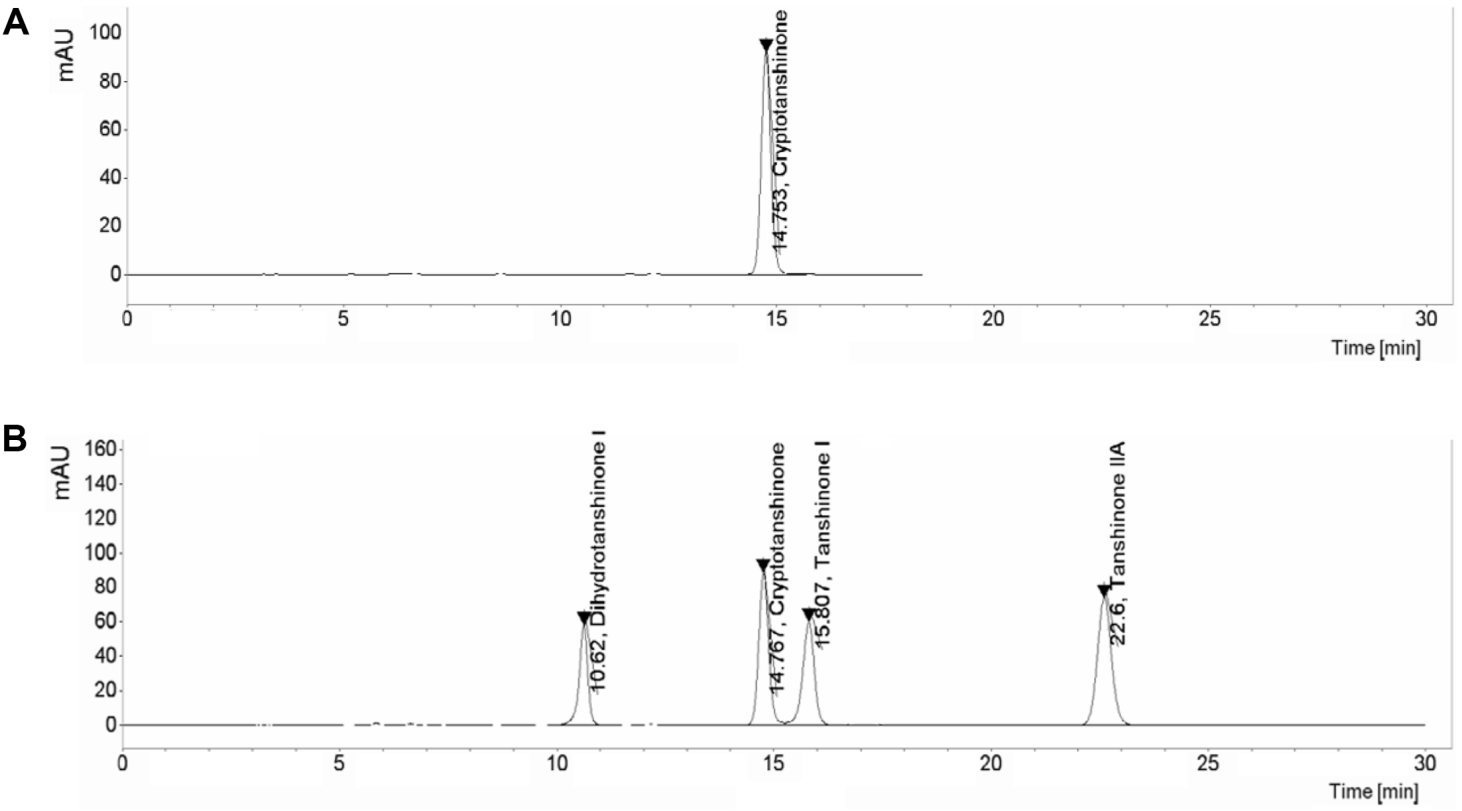
HPLC analysis of standardized *S. miltiorrhiza* Bunge extract (HLT-101). (**A**) Chromatogram of cryptotanshinone standard and (**B**) HPLC profile of major compound of HLT-101 (dihydrotanshinone I, cryptotanshinone, tanshinone I, and tanshinone IIA) in standardized HLT-101.

The results of measuring the major active compounds of HLT-101 were as follows; cryptotanshinone, tanshinine I, tanshinone IIA, and dihydrotanshinone (Fig. 1). Among them, cryptotanshinone exhibited the highest inhibitory effect on PSA expression in HLT-101. The peak of cryptotanshinone which is the most abundant component (10.25%) of HLT-101 was observed at a retention time of 14.767 min in the HPLC chromatogram (Fig. 1).

### Antioxidant Activity of HLT-101

**Fig. 2:**
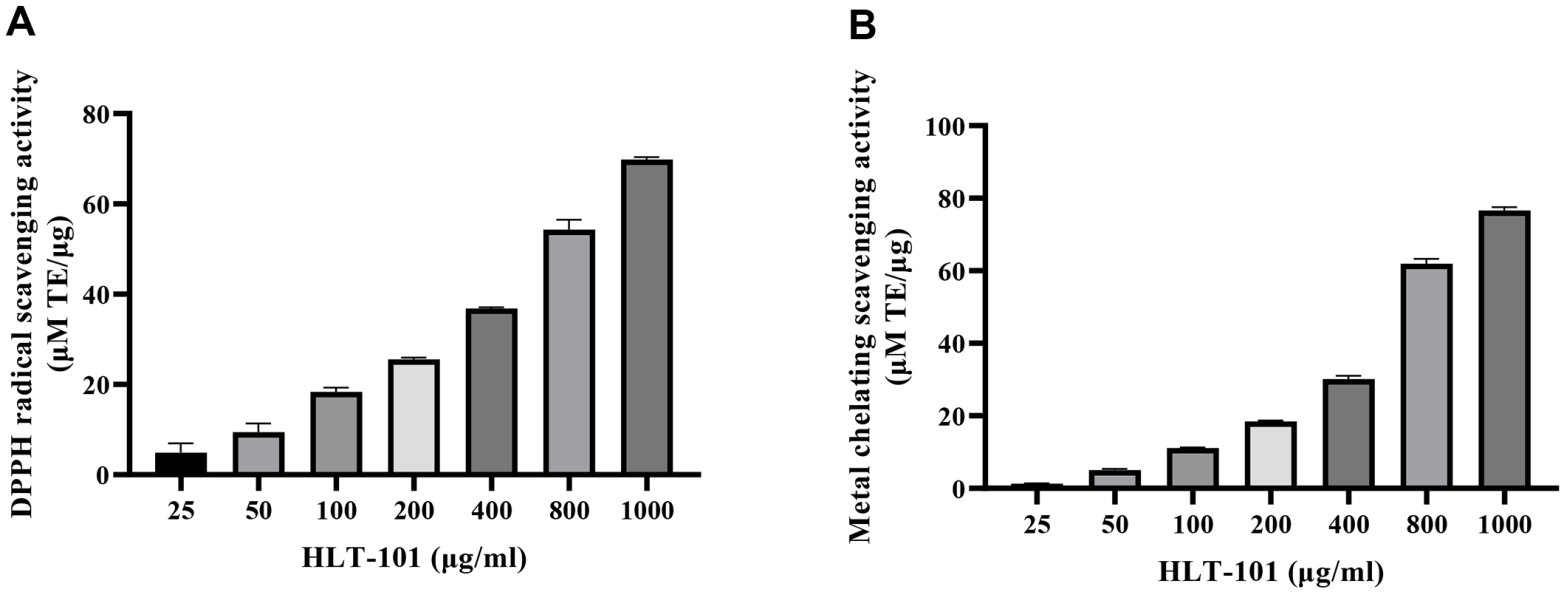
Antioxidant activities of HLT-101. (**A**) DPPH radical scavenging activity and (**B**) metal chelating scavenging activity.

Fig. 2 shows the DPPH and metal chelate scavenging activities of HLT-101. DPPH and metal chelating scavenging activity of HLT-101 expressed as IC_50_ were 675.98 and 644.60 μM TE/μg, respectively. These results indicate that HLT-101 possess strong antioxidant properties.

### Effect of HLT-101 Administration on Prostate in the BPH Rat Model

**Fig. 3:**
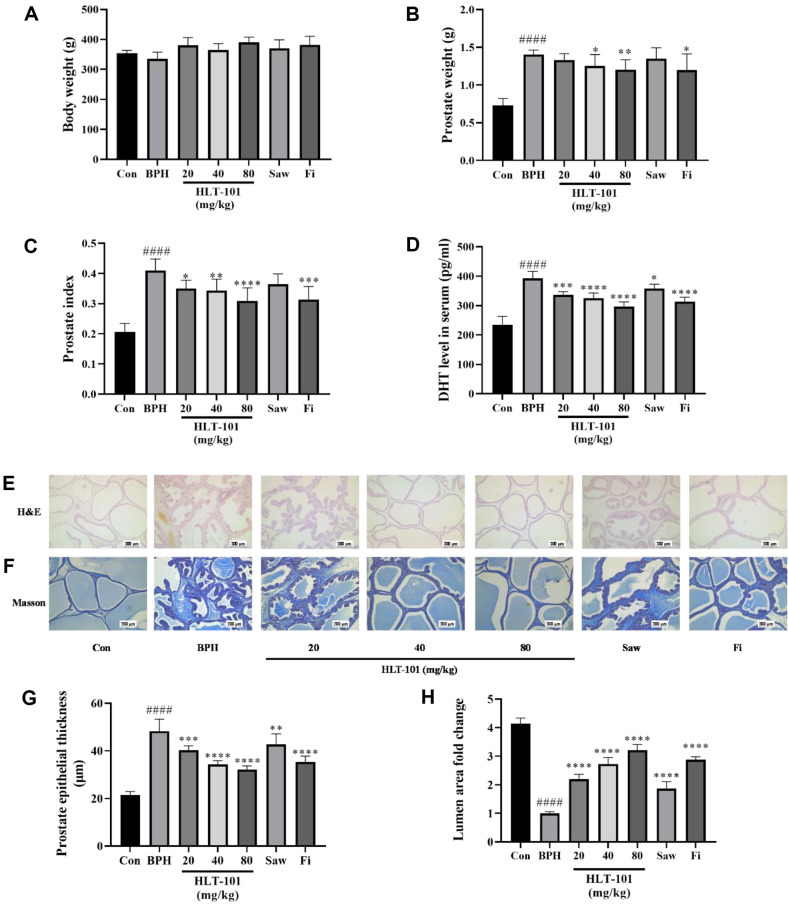
Effect of HLT-101 administration on prostate tissue in testosterone propionate (**TP**)-induced benign prostatic hyperplasia (**BPH**) rats. (**A**) Body weight. (**B**) Total prostate tissue weight and (**C**) prostate index of the rats. (**D**) DHT level in serum. (**E**) Hematoxylin and eosin-stained prostate tissues (magnification 100×). (**F**) Masson's trichrome-stained prostate tissues (magnification 50×). Prostatic smooth muscle cells were stained red; collagen fibers were stained blue. (**G**) Epithelial thickness of the prostate tissues. (**H**) Lumen area fold in the prostate tissues. Con, corn oil, subcutaneous injection (s.c.), and distilled water peroral (p.o.); BPH, TP (3 mg/kg, s.c.), and distilled water (p.o.); BPH + HLT-101, TP (3 mg/kg, s.c.), and HLT-101 (20, 40, or 80 mg/kg, p.o.); BPH + saw palmetto extract (Saw), TP (3 mg/kg, s.c.), and Saw (100 mg/kg, p.o.); BPH + finasteride (Fi), TP (3 mg/kg, s.c.), and Fi (1 mg/kg, p.o.). Data are expressed as the means ± SEMs (*n* = 8 per group). ####*p* < 0.0001, compared with the Con group, *p < 0.05, **p < 0.01, ***p < 0.001, ****p < 0.0001 compared with the BPH group.

To determine whether HLT-101 alleviated BPH symptoms, rats treated with TP were used as experimental models. As shown in Fig. 3A-3C, the prostate size of those with BPH group was significantly larger than that of those in the Con group. However, it should be noted that the BPH group that received HLT-101 demonstrated a concentration-dependent reduction in prostate weight when compared to the BPH group. The prostate index (g-prostate weight/100 g-body weight) indicated that HLT-101 was less effective than Fi, although the difference was not statistically significant. As examined by hematoxylin and eosin staining, BPH exhibited less luminal area and increased prostate epithelial tissue. Administration of HLT-101 to the BPH group suppressed epithelial tissue hyperproliferation and increased the luminal area, similar to the results observed in the Con group (Fig. 3E, 3G, 3H). The epithelial layer is supported by a fibromuscular matrix called the stroma in the prostate. The prostate stroma is composed of fibroblasts and smooth muscle cells as well as collagen, proteoglycans, and extracellular matrix (ECM). Connective tissue components are labelled using Masson's trichrome staining. Smooth muscle cells are identified by the red colour, while collagen fibers are identified by the blue colour (Fig. 3F). There was an increased amount of fibrous connective tissue in the BPH group compared to that in the Con group (Fig. 3F), and fibrosis was observed in the prostate in the BPH group and not in the Con group. The HLT-101 treatment, however, produced improvements in fibrosis in the prostate, similar to those seen in the Con group, which were similar to those seen in the Fi group. The results showed that HLT-101 treatment reduced prostate fibrosis. This indicates that HLT-101 may be an effective treatment option for BPH.

### Effects of HLT-101 on AR Signaling in BPH

**Fig. 4:**
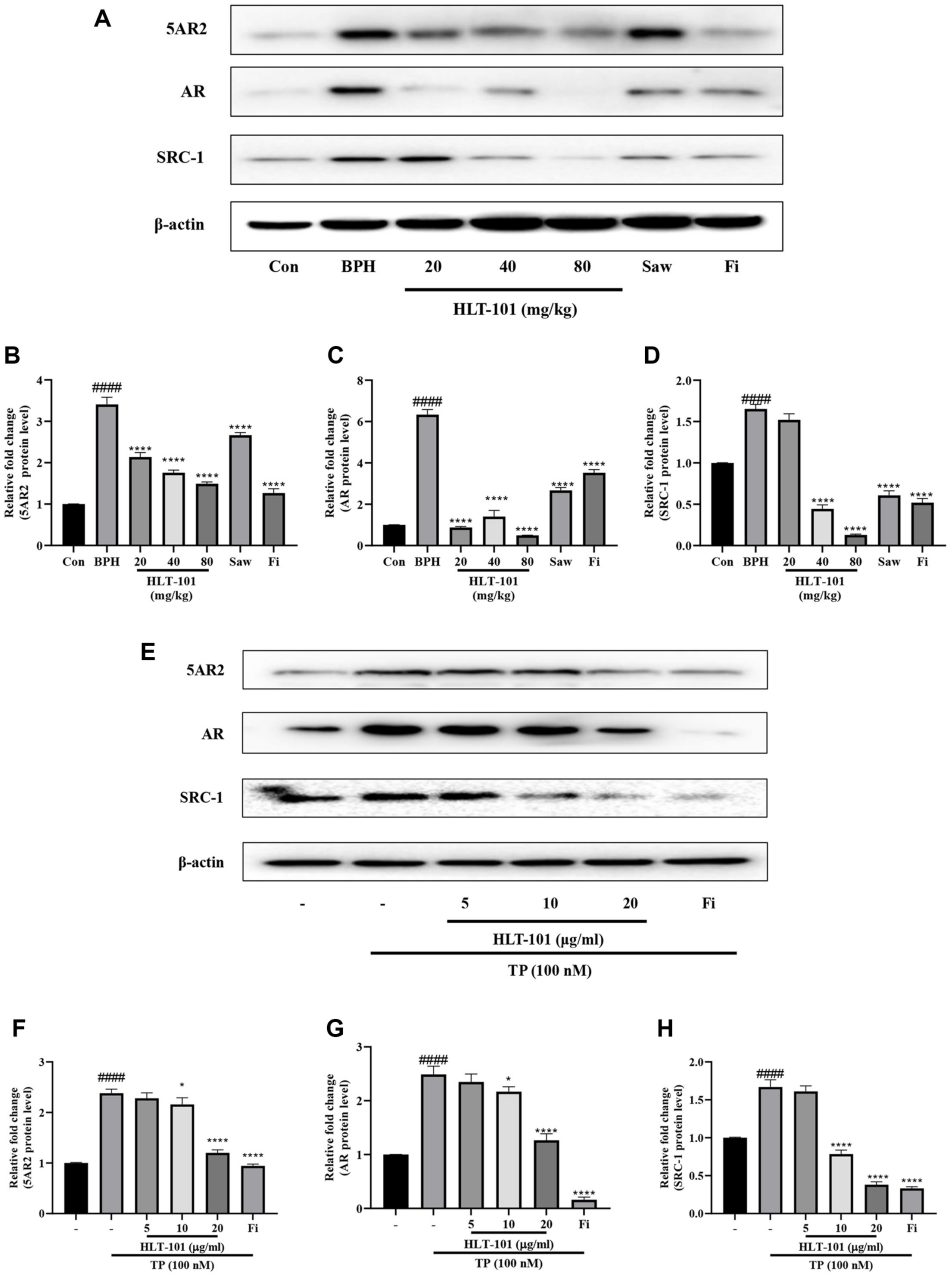
Effect of HLT-101 on androgen receptor (**AR**) signaling. (**A**) Western blot showing expression of AR signaling in prostate tissues of rat. Quantitative histogram of protein bands for the expression of (**B**) 5AR2, (**C**) AR and (**D**) SRC-1 in prostate tissues of rat. Con, corn oil, subcutaneous injection (s.c.), and distilled water peroral (p.o.); BPH, TP (3 mg/kg, s.c.), and distilled water (p.o.); BPH + HLT-101, TP (3 mg/kg, s.c.), and HLT-101 (20, 40, or 80 mg/kg, p.o.); BPH + saw palmetto extract (Saw), TP (3 mg/kg, s.c.), and Saw (100 mg/kg, p.o.); BPH + finasteride (Fi), TP (3 mg/kg, s.c.), and Fi (1 mg/kg, p.o.). Data are expressed as the means ± SEMs (*n* = 8 per group). ####*p* < 0.0001, compared with the Con group, ****p < 0.0001 compared with the BPH group. (**E**) Western blot showing expression of AR signaling in LNCaP cells. Quantitative histogram of protein bands for the expression of (**F**) 5AR2, (**G**) AR and (**H**) SRC-1 in LNCaP cells. LNCaP cells were incubated for 24 h in culture medium containing TP (100 nM), HLT-101 (5, 10, or 20 μg/ml), or Fi (1 μg/ml). Data are expressed as the means ± SEMs (*n* = 8 per group). ####*p* < 0.0001, compared with the Con group, *p < 0.05, **p < 0.01, ***p < 0.001, ****p < 0.0001 compared with the BPH group.

The inhibitory effect of HLT-101 on AR signaling was confirmed in rat tissues and LNCaP cells, an androgen-dependent prostate cancer cell line. The expression of 5AR2, AR, and SRC-1 increased after BPH induction in rats and decreased after treatment with HLT-101 (Fig. 4A-4D). We also compared the effects of HLT-101 (5, 10, and 20 μg/ml) on LNCaP cells. We examined whether HLT-101 inhibits AR signaling when TP is applied to LNCaP cells. When LNCaP cells were treated with TP, the expression of 5AR2, AR, and SRC-1 significantly increased compared to that in the untreated group. Treatment of LNCaP cells with HLT-101 inhibited the expression of 5AR2, AR, and SRC-1 in a concentration-dependent manner (Fig. 4E-4H). Interestingly, HLT-101 suppressed the expression of AR signaling-related proteins. These results suggest that HLT-101 inhibits AR signaling by suppressing the expression of AR-related proteins. Thus, HLT-101 may be a potential therapeutic agent for the treatment of BPH.

### Effects of HLT-101 on Protein Expression of Growth Factor in BPH

**Fig. 5:**
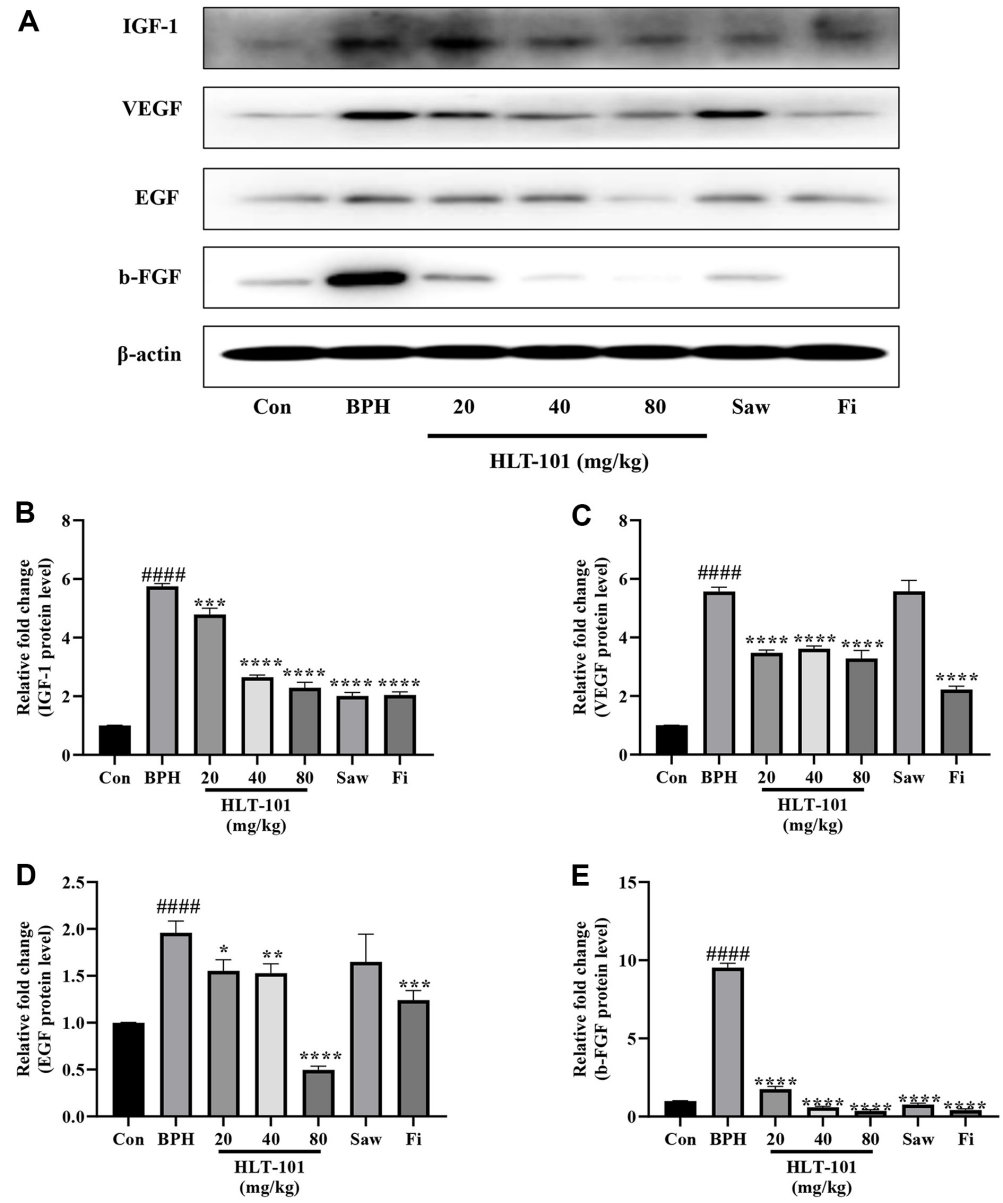
Effect of HLT-101 on protein expression of growth factor in TP-induced BPH rats. (**A**) Western blot showing expression of growth factor in prostate tissues of rat. Quantitative histogram of protein bands for the expression of (**B**) IGF-1, (**C**) VEGF, (**D**) SRC-1 and (**E**) b-FGF in prostate tissues of rat. Con, corn oil, subcutaneous injection (s.c.) and distilled water peroral (p.o.); BPH, TP (3 mg/kg, s.c.), and distilled water (p.o.); BPH + HLT-101, TP (3 mg/kg, s.c.), and HLT-101 (20, 40, or 80 mg/kg, p.o.); BPH + saw palmetto extract (Saw), TP (3 mg/kg, s.c.), and Saw (100 mg/kg, p.o.); BPH + finasteride (Fi), TP (3 mg/kg, s.c.), and Fi (1 mg/kg, p.o.). Data are expressed as the means ± SEMs (*n* = 8 per group). ####*p* < 0.0001, compared with the Con group, *p < 0.05, **p < 0.01, ***p < 0.001, ****p < 0.0001 compared with the BPH group.

As shown in Fig. 4, AR signaling was inhibited by HLT-101 treatment. AR signaling is known to be involved in and upregulate the expression of growth factors. Therefore, it was confirmed that HLT-101 suppressed growth factors expression in the prostate. The BPH group exhibited significantly higher growth factor protein levels than the Con group (Fig. 5). The HLT-101 treatment group, however, demonstrated a significant decline in growth factor levels in comparison to the BPH group. Overall, these findings suggest that AR signaling is involved in the expression of growth factors, and that HLT-101 is able to reduce their expression in BPH.

### Effects of HLT-101 on Inflammation and Nrf-2 Expression in BPH

**Fig. 6:**
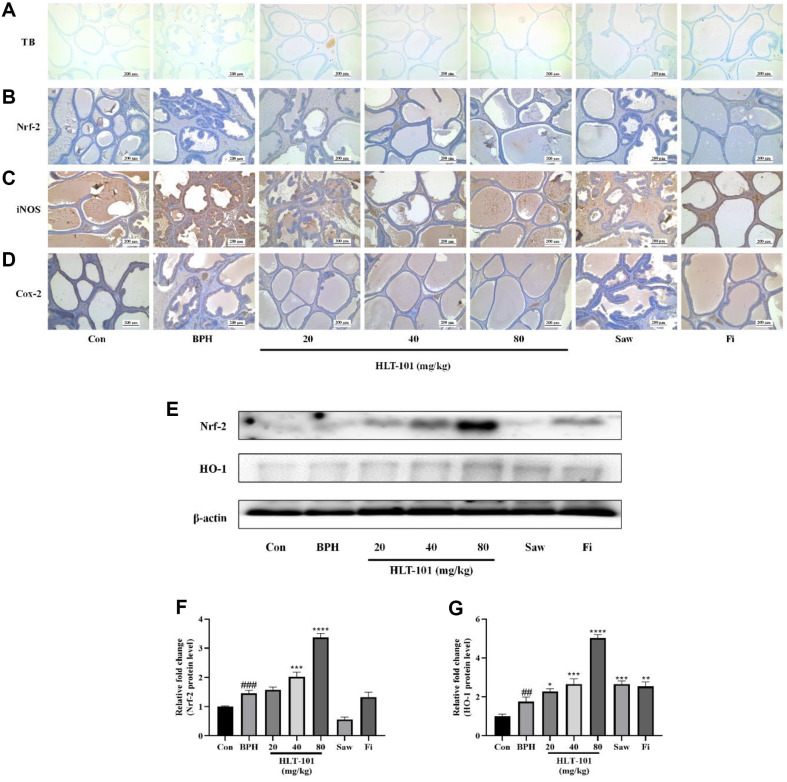
Effect of HLT-101 on the expression of Nrf-2 and inflammatory proteins in TP-induced BPH rats. (**A**) Toluidine blue staining. (**B**) Immunohistochemical staining images of Nrf-2, (**C**) iNOS, and (**D**) Cox-2. (**E**) Western blot showing expression of Nrf-2/HO-1 signaling in prostate tissues of rat. Quantitative histogram of protein bands for the expression of (**F**) Nrf-2 and (**G**) HO-1 in prostate tissues of rat. Con, corn oil, subcutaneous injection (s.c.) and distilled water peroral (p.o.); BPH, TP (3 mg/kg, s.c.) and distilled water (p.o.); BPH + HLT-101, TP (3 mg/kg, s.c.), and HLT-101 (20, 40, or 80 mg/kg, p.o.); BPH+ saw palmetto extract (Saw), TP (3 mg/kg, s.c.), and Saw (100 mg/kg, p.o.); BPH + finasteride (Fi), TP (3 mg/kg, s.c.), and Fi (1 mg/kg, p.o.). Data are expressed as the means ± SEMs (*n* = 8 per group). #*p* < 0.05, ##*p* < 0.01, compared with the Con group, *p < 0.05, **p < 0.01, ****p < 0.0001 compared with the BPH group.

The toluidine blue staining selectively stains mast cells, allowing them to be identified and distinguished from other cell types. It was confirmed by toluidine blue staining that mast cells were present within stromal cells (Fig. 6A). It was found that the number of mast cells was higher in the BPH group than in the Con group. The mast cell infiltration in the HLT-101 group was lower than that in the BPH group. According to western blot analysis, Nrf-2 expression decreased in the BPH group compared to the Con group (Fig. 6B), in agreement with immunohistochemistry (Fig. 6E). In contrast, in the group treated with 40 or 80 mg/kg HLT-101, Nrf-2 expression was elevated in the prostate tissue in a concentration-dependent manner (Fig. 6B). The expression of HO-1, a subfactor of Nrf-2, also increased in the HLT-101-treated group in a concentration-dependent manner relative to the BPH group. Immunohistochemistry results to determine the expression of inflammatory cytokines such as Cox-2 and iNOS showed that the expression of Cox-2 and iNOS was greatly increased in the prostate tissue of the BPH group compared to the Con group (Fig. 6C, 6D). In contrast, the HLT-101 treatment group showed significantly reduced expression of Cox-2 and iNOS compared to that in the BPH group, indicating suppression of inflammatory cytokines in the prostate.

**Fig. 7:**
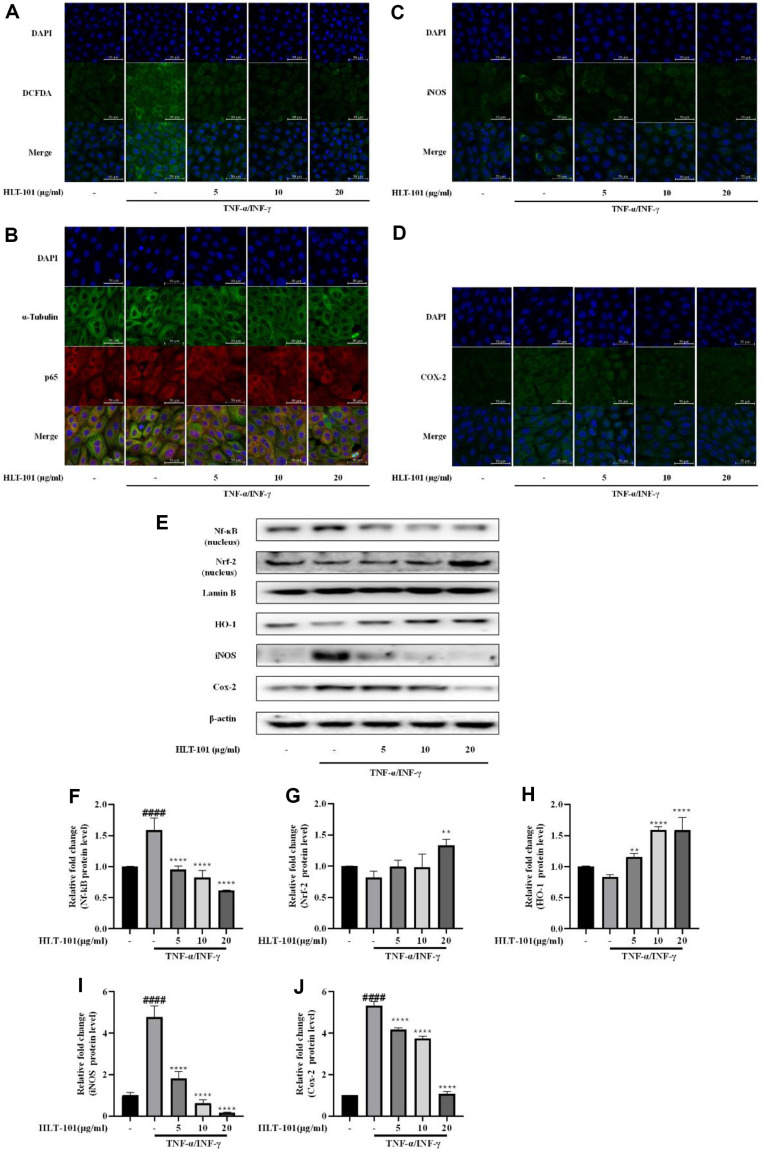
Effect of HLT-101 on the expression of Nrf-2 and inflammatory proteins in BPH-1 cells. (**A**) Fluorescence microscopic images of intracellular ROS by DCFDA assay. (**B**) Fluorescence microscopic images of NF-κB, (**C**) iNOS, and (**D**) Cox-2. (**E**) Western blot showing expression of NF-κB and Nrf-2/HO-1 in BPH-1 cells. Quantitative histogram of protein bands for the expression of (**F**) NF-κB, (**G**) Nrf-2, (**H**) HO-1, (**I**) iNOS and (**J**) Cox-2 in BPH-1 cells. BPH-1 cells were incubated for 6 h in culture medium containing TNF-α (10 ng/ml), INF-γ (10 ng/ml) and HLT-101 (5, 10, or 20) μg/ml. Data are expressed as the means ± SEMs (*n* = 8 per group). ####*p* < 0.0001, compared with the Con group, *p < 0.05, **p < 0.01, ***p < 0.001, ****p < 0.0001 compared with the BPH group.

To confirm whether HLT-101 activates Nrf-2 and reduces inflammatory factors in an inflammatory situation, BPH-1 cells were treated with both TNF-α and IFN-γ. ROS levels were increased in BPH-1 cells co-treated with both TNF-α and IFN-γ. In contrast, treatment with HLT-101 significantly reduced intracellular ROS levels (Fig. 7A). When BPH-1 cells were treated with TNF-α and IFN-γ, it was confirmed that NF-κB translocated to the nucleus and was activated. In contrast, treatment with HLT-101 inhibited the activation of NF-κB (Fig. 7B, 7E, 7F). The expression of iNOS and Cox-2 was also significantly reduced in cells treated with HLT-101 compared to the negative Con group (Fig. 7C, 7D). Treatment with HLT-101 inhibited the expression of inflammatory factors by activating the Nrf-2 pathway and inhibiting the activity of NF-κB in an inflammatory environment (Fig. 7E-7J). This suggests that HLT-101 is a potential therapeutic agent for inflammatory diseases. HLT-101 may also be useful in reducing inflammation in BPH.

### Effects of HLT-101 on Proliferation and Apoptosis in BPH

**Fig. 8:**
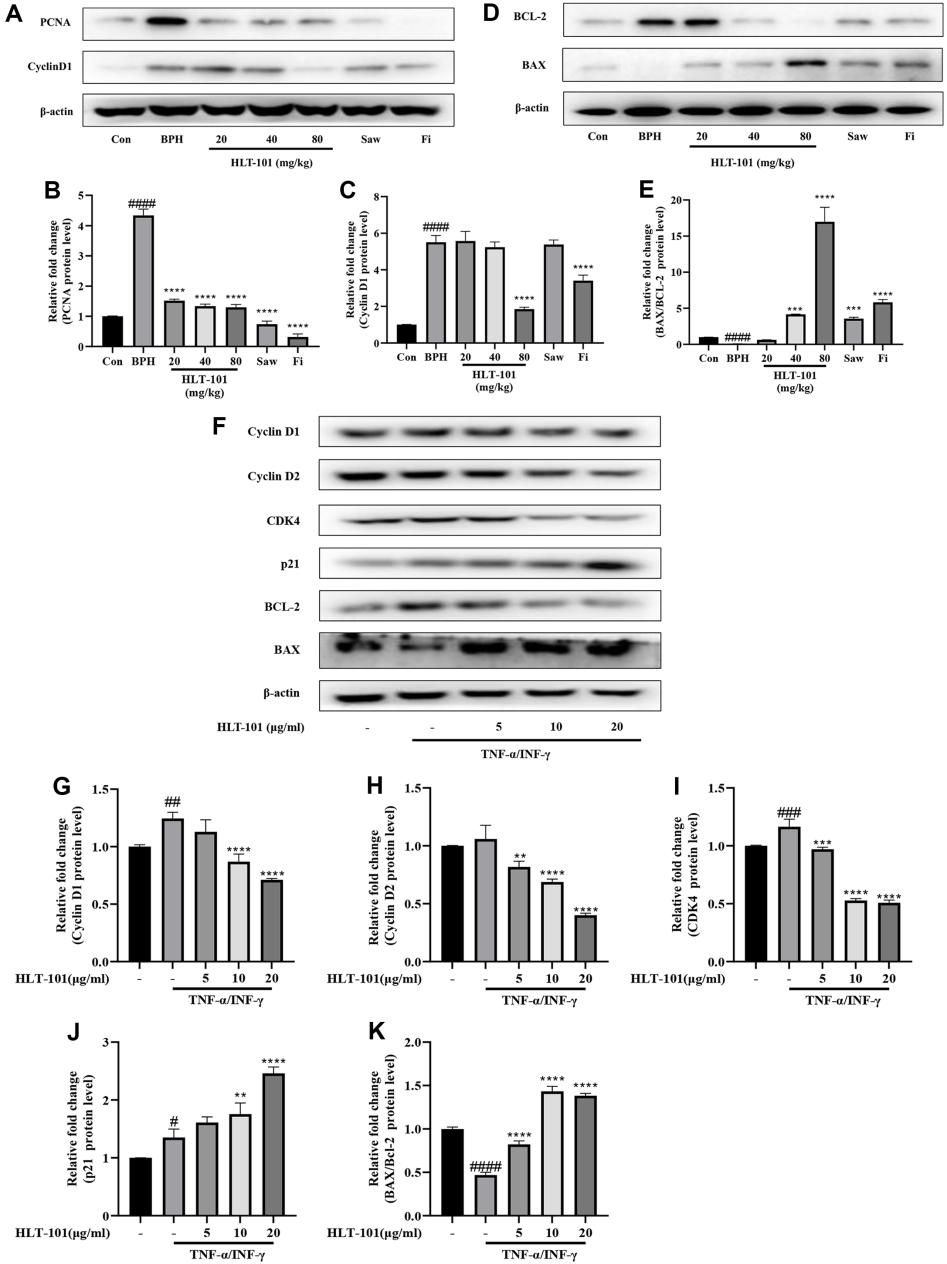
Effects of HLT-101 on proliferation and apoptosis in BPH. (**A**) Western blot showing expression of proliferation-related proteins in prostate tissues of rat. Quantitative histogram of protein bands for the expression of (**B**) PCNA, (**C**) Cyclin D1 in prostate tissues of rat. (**D**) Western blot showing expression of apoptosis-related proteins in TP-induced BPH rats. Quantitative histogram of protein bands for the expression of (**E**) BAX/BCL-2 in prostate tissues of rat. Con, corn oil, subcutaneous injection (s.c.) and distilled water peroral (p.o.); BPH, TP (3 mg/kg, s.c.) and distilled water (p.o.); BPH + HLT- 101, TP (3 mg/kg, s.c.), and HLT-101 (20, 40, or 80 mg/kg, p.o.); BPH + saw palmetto extract (Saw), TP (3 mg/kg, s.c.), and Saw (100 mg/kg, p.o.); BPH + finasteride (Fi), TP (3 mg/kg, s.c.), and Fi (1 mg/kg, p.o.). Data are expressed as the means ± SEMs (*n* = 8 per group). ####*p* < 0.0001, compared with the Con group, ***p < 0.001, ****p < 0.0001 compared with the BPH group. (**F**) Western blot showing expression of proliferation- and apoptosis-related proteins in BPH-1 cells. Quantitative histogram of protein bands for the expression of (**G**) Cyclin D1, (**H**) Cyclin D2, (**I**) CDK4, (**J**) p21 and (**K**) BAX/BCL-2 in BPH-1 cells. BPH- 1 cells were incubated for 6 h in culture medium containing TNF-α (10 ng/ml), INF-γ (10 ng/ml) and HLT-101 (5, 10, or 20) μg/ ml. Data are expressed as the means ± SEMs. #*p* < 0.05, ##*p* < 0.01, ###*p* < 0.001, ####*p* < 0.0001, compared to untreated cells, **p < 0.01, ***p < 0.001, ****p < 0.0001 compared with TNF-α/INF-γ treated cells.

AR signaling and expression of cell proliferation-related factors via the AR signaling and NF-κB pathway contribute to BPH development. HLT-101 targets the AR signaling and NF-κB pathway, which are responsible for the expression of cell proliferation-related factors. By inhibiting AR signaling and inflammation, HLT-101 is able to prevent this imbalance between cell proliferation and apoptosis, thus preventing BPH development. We confirmed the expression of proliferation and apoptosis-related factors in HLT-101-treated BPH rat model and BPH-1 cell lines. As compared to the Con group, PCNA and cyclin D1 were overexpressed in the BPH rat model (Fig. 8A-8C). In this study, HLT-101 was shown to inhibit BPH cell proliferation. In BPH-induced rats, HLT-101 downregulated BCL-2 expression, which inhibits apoptosis, and upregulated that of BAX expression, which promotes apoptosis (Fig. 8D, 8E). To further verify the effect of HLT-101 on the proliferation of BPH-1 cells, we quantified the protein expression of cyclin D1, cyclin D2 and CDK4, p21. TNF-α and IFN-γ treatment of BPH-1 cells significantly increased the expression of cyclin D1, cyclin D2, and CDK4 compared to that in the untreated group (Fig. 8F-8I). HLT-101 suppressed cyclin and CDK expression compared to the TNF-α and IFN-γ treatment groups. Contrastively, the expression of anti-proliferation factor p21 was increased by the HLT-101 in a dose dependent manner (Fig. 8F and 8J). Moreover, HLT-101 inhibited the expression of anti-apoptotic proteins, such as BCL-2 and upregulated BAX, suggesting that HLT-101 stimulated apoptosis (Fig. 8F and 8K). This suggests that HLT-101 induced apoptosis by reducing the expression of proteins involved in the cell cycle. In the TNF-α and IFN-γ treated cells, the expression of cyclins and CDKs was increased; however, in the presence of HLT-101, these proteins were suppressed, which in turn inhibited the expression of anti-apoptotic proteins. This indicates that HLT-101 induced apoptosis in BPH-1 cells.

## Discussion

BPH is a widespread and chronic progressive disease characterized by prostatic hyperplasia with stromal and glandular cell hyperplasia [36]. Age is considered the most critical risk factor for BPH because its incidence increases with age, particularly after 50 years [37]. Additionally, some studies have suggested that chronic inflammation, oxidative stress, and sex hormone imbalance may contribute to the development of BPH. Inflammation can increase ROS production and deplete antioxidant protective systems [38]. All of these risk factors may be linked to the fact that they can lead to increased oxidative stress, a major cause of BPH development.

Oxidative stress occurs when there is an imbalance between free radical production and the antioxidant protection system, resulting in cellular damage [39]. This can lead to increased inflammation and contribute to the development of BPH. Clinical studies have confirmed reduced function of antioxidant defense systems in elderly patients [40]. Other studies have also shown that oxidative stress-mediated pathways are involved in several male urinary disorders [41]. As a matter of fact, testosterone injections have been demonstrated to induce excessive production of free radicals and to weaken cellular antioxidant mechanisms in BPH models [26]. Additionally, Nrf-2/HO-1 plays a crucial role in antioxidant balance and inflammation, which has been linked to BPH pathogenesis [42]. The Nrf-2/NF-κB signaling pathway and the AR signaling pathway are closely connected. It has been shown that Nrf-2 activation inhibits AR signaling and NF-κB activation [43, 44]. As well as promoting the expression of Cox-2 and iNOS, testosterone also promotes the expression of other inflammatory proteins. Aside from this, there is considerable evidence that NF-κB is involved in the regulation of cell growth and proliferation [45]. Therefore, increased inflammation due to oxidative stress may lead to increased NF-κB activity, contributing to BPH development. This increased NF-κB activity can, in turn, lead to higher levels of BPH-related gene expression, inflammation, and cell proliferation. This, in turn, can lead to an increase in the prostate size, which is a symptom of BPH. Additionally, Nrf-2/HO-1 activation can also reduce the activation of AR signaling, which is known to be associated with BPH pathogenesis [20, 46]. As a result, Nrf-2/HO-1 activation could potentially reduce inflammation and oxidative stress, thereby ameliorating the symptoms of BPH.

The results of this study also confirmed that testosterone administration increased the prostate tissue size compared to that of the Con group. Compared to the Con group, the expression of androgen signaling, growth factors, and inflammatory factors such as Cox-2 and iNOS, was upregulated in the BPH group, whereas the expression of Nrf-2 and HO-1 was downregulated. Previous studies also confirmed the marked decrease in expression levels of Nrf-2 and HO-1 in prostatic tissue of testosterone treated rat [26, 42]. The HLT-101 treatment significantly overexpressed Nrf-2 and HO-1. In addition, it inhibited the expression of activated AR signals, growth factors, and inflammatory fac-tors, thereby reducing prostate size in a concentration-dependent manner. HLT-101 is believed to activate Nrf-2 to suppress inflammatory cytokine levels and testosterone-induced ROS production, thereby inhibiting prostate cancer cell proliferation. This suggests that HLT-101 may attenuate the adverse effects of testosterone on prostate tissue by reducing the expression of AR signaling and NF-κB activation while increasing antioxidant and anti-inflammatory responses in the prostate. Additionally, HLT-101 reduced the expression of growth factors in the prostate tissue. This suggests that HLT-101 suppresses AR signaling, thereby reducing the expression of growth factors. In addition to the reduction in growth factor expression due to AR signaling following HLT-101 administration, inflammation relief may have contributed to the balance between cell proliferation and apoptosis.

During the course of the testosterone injection, the balance between cell proliferation and death is disrupted, which can lead to cell proliferation and prostate enlargement. Injection of testosterone increases PCNA levels in prostate cells. This increase in PCNA levels leads to an increase in the cell cycle rate, resulting in an increase in the number of cells. This increase in cell number results in prostate enlargement. The results of this study confirmed that the expression of PCNA and cyclin D1 was inhibited by HLT-101 treatment. HLT-101 treatment inhibited the expression of PCNA and cyclin D1, which are both re-sponsible for the increase in the cell cycle rate. As a result, the number of cells did not increase and the prostate was not enlarged. On the other hand, it has been shown that BAX, one of the pro-apoptosis genes, can induce the release of cytochrome c (cyt c), which in turn triggers apoptosis [47]. As the name suggests, BCL-2 plays a role in preventing mitochondria from releasing cyt c [48]. Consequently, the ratio of BAX/BCL-2 is regarded as one of the most important indicators of the apoptotic process. When the ratio of BAX/BCL-2 is high, BCL-2 is not able to prevent the release of cyt c, which allows for the apoptotic process to begin. This means that the higher the BAX/BCL-2 ratio, the more likely it is that apoptosis will occur. In this research, HLT-101 induced apoptosis in prostate cancer cells by downregulating the expression of BCL-2, an apoptosis inhibitor, and up-regulating BAX, a pro-apoptotic factor. By inhibiting the expression of PCNA and cyclin D1, HLT-101 was able to slow the cell cycle, preventing cell division and reducing cell number. Additionally, HLT-101 induced apoptosis by downregulating BCL-2 and upregulating BAX, two factors involved in apoptosis. Moreover, HLT-101 was able to achieve these effects without causing any significant toxicity to the cells, demonstrating its potential as an effective therapeutic agent.

This study demonstrated that HLT-101 suppressed AR signaling, alleviated testosterone-induced inflammation in the prostate, and activated Nrf-2/HO-1 signaling. It was confirmed that the treatment of TNF-α and IFN-γ in BPH-1 increased intracellular ROS levels, activated NF-κB, and reduced the activity of Nrf-2 where treatment of HLT-101 decreased the TNF-α/IFN-γ-induced ROS levels through the activation of Nrf-2.

## Conclusion

Oxidative stress induced cellular damage leading to inflammation has been identified as one of the causative factors of the development of BPH. Further, activation of Nrf-2/HO-1 has played a key role in controlling pathogenesis of BPH leading to reduced levels of ROS. Subsequently, the current study confirmed that HLT-101 treatment de-creased the intracellular ROS level in BPH-1 cells through the activation of Nrf-2. Moreover, HLT-101 suppressed AR signaling and NF-κB activation, alleviated testosterone-induced prostate inflammation. Therefore, the study confirmed the potential utilization of HLT-101 in the treatment of BPH.
